# Correction: Design and fabrication of a microfluidic chip to detect tumor markers

**DOI:** 10.1039/d0ra90140g

**Published:** 2021-01-22

**Authors:** Cuimin Sun, Hui You, Nailong Gao, Jianguo Chang, Qingxue Gao, Yang Xie, Yao Xie, Ronald X. Xu

**Affiliations:** Department of Mechanical Engineering and Precision Machinery, University of Science and Technology of China Hefei Anhui 230001 PR China; XingJian College of Science and Liberal Arts of Guangxi University Nanning Guangxi 530004 PR China; Anhui Huiwei Biotechnology Co., Ltd. PR China; Department of Mechanical Engineering, Guangxi University Nanning Guangxi 530004 PR China hyou@gxu.edu.cn

## Abstract

Correction for ‘Design and fabrication of a microfluidic chip to detect tumor markers’ by Cuimin Sun *et al.*, *RSC Adv.*, 2020, **10**, 39779–39785, DOI: 10.1039/D0RA06693A.

The authors regret that there was an affiliation missing in the original article for the author Cuimin Sun. The corrected and complete list of author affiliations is given in this document.

The Royal Society of Chemistry apologises for these errors and any consequent inconvenience to authors and readers.

## Supplementary Material

